# Biological sex modifies the link between nutrient intake and metabolic dysfunction–associated steatotic liver disease: intrinsic oxidative vulnerability in females

**DOI:** 10.1210/jendso/bvag253

**Published:** 2026-10-27

**Authors:** Alina Khusnullina, Hisanori Goto, Kumi Kimura, Alisa Arslanova, Hiroaki Takayama, Yujiro Nakano, Yumie Takeshita, Hiromasa Tsujiguchi, Akinori Hara, Hiroyuki Nakamura, Kenichi Harada, Yasuhiko Yamamoto, Toshinari Takamura

**Affiliations:** Department of Endocrinology and Metabolism, Kanazawa University Graduate School of Medical Sciences, Ishikawa 920-8640, Japan; Department of Endocrinology and Metabolism, Kanazawa University Graduate School of Medical Sciences, Ishikawa 920-8640, Japan; Department of Biochemistry and Molecular Vascular Biology, Kanazawa University Graduate School of Medical Sciences, Ishikawa 920-8640, Japan; Department of Biochemistry and Molecular Vascular Biology, Kanazawa University Graduate School of Medical Sciences, Ishikawa 920-8640, Japan; Department of Endocrinology and Metabolism, Kanazawa University Graduate School of Medical Sciences, Ishikawa 920-8640, Japan; Life Sciences Division, Engineering and Technology Department, Kanazawa University Graduate School of Medical Sciences, Ishikawa 920-8640, Japan; Department of Endocrinology and Metabolism, Kanazawa University Graduate School of Medical Sciences, Ishikawa 920-8640, Japan; Department of Endocrinology and Metabolism, Kanazawa University Graduate School of Medical Sciences, Ishikawa 920-8640, Japan; Department of Hygiene and Public Health, Faculty of Medicine, Institute of Medical, Pharmaceutical and Health Sciences, Kanazawa University, Ishikawa 920-8640, Japan; Department of Hygiene and Public Health, Faculty of Medicine, Institute of Medical, Pharmaceutical and Health Sciences, Kanazawa University, Ishikawa 920-8640, Japan; Department of Hygiene and Public Health, Faculty of Medicine, Institute of Medical, Pharmaceutical and Health Sciences, Kanazawa University, Ishikawa 920-8640, Japan; Department of Human Pathology, Kanazawa University Graduate School of Medical Sciences, Ishikawa 920-8640, Japan; Department of Biochemistry and Molecular Vascular Biology, Kanazawa University Graduate School of Medical Sciences, Ishikawa 920-8640, Japan; Department of Endocrinology and Metabolism, Kanazawa University Graduate School of Medical Sciences, Ishikawa 920-8640, Japan

**Keywords:** MASLD, fibrosis, Western diet, sex differences, Kupffer cells, glutathione metabolism

## Abstract

**Purpose:**

Whether biological sex modifies the association between diet and liver disease remains unclear. We applied a reverse-translational strategy linking sex-stratified population data with mechanistic studies in a murine model.

**Methods:**

We analyzed associations between dietary nutrients and hepatic steatosis in a community-based Japanese cohort (the Shika study). Based on the human findings, mice were fed a Western diet with fructose–sucrose–supplemented water to investigate sex-dependent mechanisms.

**Results:**

In the Shika study, steatosis in women—but not in men—was associated with higher dietary cholesterol/fat density and with a higher sucrose-to-total carbohydrate ratio. Modeling this risk in mice revealed a dissociation between systemic metabolism and liver pathology: despite exhibiting milder systemic obesity than males, females developed more pronounced fibrosis and oxidative injury. Transcriptomic analysis identified distinct sex-biased pathways. Males preferentially upregulated genes involved in glutathione metabolism (eg, *Gst* and *Gpx*). In contrast, females exhibited higher expression of genes associated with microsomal ω-oxidation and acyl-CoA hydrolysis (eg, *Cyp4a* and *Acot*), accompanied by marked oxidative stress and a Kupffer cell–skewed inflammatory signature.

**Conclusion:**

By integrating sex-stratified epidemiological evidence in humans with the intrinsic oxidative vulnerability identified in mice, our findings highlight biological sex as a critical modifier of nutrition-driven liver injury. These findings suggest that rigorous nutritional management is particularly important for preventing disease progression in women with metabolic dysfunction–associated steatotic liver disease.

Metabolic dysfunction–associated steatotic liver disease (MASLD) has emerged as a major global health challenge and is a key driver of the rising incidence of cirrhosis and hepatocellular carcinoma (1). Although metabolic dysregulation underpins MASLD pathogenesis, the marked heterogeneity in liver outcomes among individuals exposed to similar dietary environments suggests the presence of critical modifying factors. Biological sex is a well-recognized contributor to this heterogeneity (2-5), yet how specific nutrient patterns interact with sex to influence liver pathology remains poorly understood. Moreover, many mechanistic studies rely on hepatotoxic agents that induce rapid and artificial injury, which does not adequately model the chronic, nutrition-driven pathogenesis of human MASLD (6, 7). Consequently, experimental systems that more faithfully reflect the specific dietary risk patterns observed in human populations are needed.

In this study, we applied a reverse-translational approach that links epidemiological signals directly to mechanistic inquiry. First, we identified sex-specific associations between nutrient intake and liver steatosis in the Shika study, a community-based Japanese cohort. Although this epidemiological analysis provided robust evidence of diet-associated steatosis, the reliance on noninvasive imaging limited the ability to assess more advanced stages of liver disease in the human cohort. To address this limitation and capture the full spectrum of disease progression, we designed a murine model to replicate the specific nutrient imbalances identified in humans, rather than relying on generic hypercaloric diets. Using this nutrition-driven model, we extend our findings beyond steatosis to characterize hepatic oxidative stress, inflammation, and fibrogenic responses—pathological features that are critical determinants of prognosis but are difficult to quantify in population-based studies. Finally, we integrated transcriptomic analyses to identify molecular pathways underlying sex-dependent vulnerability to these specific nutrient imbalances, thereby providing a highly relevant framework for developing sex-aware therapeutic strategies.

## Materials and methods

### Epidemiological study (Shika study)

#### Ethics

This study was conducted in accordance with the principles of the Declaration of Helsinki and was approved by the Human Studies Ethics Committee of Kanazawa University Hospital (approval numbers: 2016-376 [1491] and 2019-017 [326]). The study was registered in the University Hospital Medical Information Network Clinical Trials Registry (UMIN000024915). Written informed consent was obtained from all participants prior to enrollment.

#### Study design and participants

Data were obtained from the Shika Study, a community-based cross-sectional health survey conducted between 2013 and 2019 in Shika Town, Ishikawa Prefecture, Japan (8, 9). Using cluster sampling, all residents aged ≥40 years who were legally registered in 2 designated elementary school districts (Horimatsu and Higashimasuho) were invited to participate (*n* = 2160). Of these, 1335 adults completed the health examination and blood sampling and were eligible for screening.

#### Dietary assessment and energy-adjusted nutrient intakes

Habitual dietary intake over the preceding month, including alcohol consumption, was assessed using the validated Japanese version of the Brief-type Self-administered Diet History Questionnaire (BDHQ), which captures the frequency of intake of 58 commonly consumed food and beverage items (10). Daily intakes of total energy and nutrients were estimated using the standard BDHQ nutrient calculation procedure. To account for energy intake, nutrient intakes were energy-adjusted using the nutrient density method and expressed per 1000 kcal. As an exploratory analysis of carbohydrate composition, we also evaluated the sucrose-to-total carbohydrate ratio (%), calculated as sucrose intake divided by total carbohydrate intake × 100.

#### Liver steatosis assessment

Hepatic steatosis was assessed by abdominal ultrasonography as part of the health examination. Participants were classified as having liver steatosis (present/absent) based on ultrasound findings evaluated by trained personnel according to standard clinical criteria.

#### Exclusion criteria and analytic sample

Participants were sequentially excluded for the following reasons: missing BDHQ data (*n* = 271), excessive alcohol intake (*n* = 205), missing sex information (*n* = 5), missing abdominal ultrasound data (*n* = 191), and implausible total energy intake on the BDHQ (<600 or >4000 kcal/day; *n* = 6). Excessive alcohol intake was defined as >30 g/day for men and >20 g/day for women. The final analytic sample comprised 657 participants (251 men and 406 women).

#### Statistical analysis for epidemiological data

All analyses were conducted separately for men and women. Energy-adjusted nutrient intakes (per 1000 kcal) were summarized according to liver steatosis status. Baseline characteristics were compared according to ultrasound-defined liver steatosis status within each sex (Table S1). Continuous variables were compared using the Mann–Whitney *U* test, and categorical variables were compared using Fisher exact test. Because direct menopausal status was unavailable, age ≥55 years was used as an approximate proxy for postmenopausal age. In an exploratory analysis restricted to participants with ultrasound-defined liver steatosis, age and fibrosis-4 index (FIB-4) were compared between women and men. Additional multivariable logistic regression analyses were performed in women using ultrasound-defined liver steatosis as the outcome. Each nutrient variable was entered into a separate model adjusted for age, alcohol intake, total energy intake, hemoglobin A1c (HbA1c), body mass index (BMI), and smoking status. A 2-sided *P*-value <.05 was considered statistically significant.

### Animal experiments

#### Animals and ethical approval

All mouse experiments were conducted in accordance with institutional guidelines and were approved by the Animal Ethics Committee of Kanazawa University (AP23-030), as well as applicable national regulations governing animal welfare. Six-week-old male and female C57BL/6J mice were purchased from Japan SLC (Shizuoka, Japan). Mice were housed at 22 ± 2 °C under a 12-hour light/12-hour dark cycle with ad libitum access to food and water and were acclimated for at least 1 week before experimentation. Animals were group-housed (3 per cage) and randomly assigned to experimental groups.

#### Diet-induced steatohepatitis model (Western diet)

For the diet-induced steatohepatitis model, we employed a protocol based on a previous report shown to induce robust hepatic inflammation and fibrosis (11).

Mice were fed a Western diet (WD) containing 58% fat and 1.0% added cholesterol (high-fat, cholesterol diet; D09061703, Research Diets) for 12 weeks, and drinking water was supplemented with fructose (23.1 g/L) and sucrose (18.9 g/L) throughout the feeding period. This differs from previously reported model (12) with respect to fat content, cholesterol content, sugar supplementation, and feeding duration. Sample sizes were *n* = 17 male mice, *n* = 18 female mice (Fig. 1A), and *n* = 9 female mice after ovariectomy (Fig. S2A (13)). Body weight, blood glucose, and food intake were monitored during the experiment. An intraperitoneal glucose tolerance test (0.375 mg/g body weight) was performed after 10 weeks of WD feeding following a 12-hour fast, and blood was collected 30 minutes after glucose administration for insulin measurement. An insulin tolerance test (1 mU/g body weight) was performed after 11 weeks of WD feeding following a 4-hour fast. Control group mice were fed a normal chow (MF; Oriental Yeast Co., Ltd, Tokyo, Japan) with regular drinking water and sacrificed at the same age as mice fed a WD (19 weeks old). Sample sizes were *n* = 3 male and *n* = 4 female mice (Fig. 1A).

**Figure 1:**
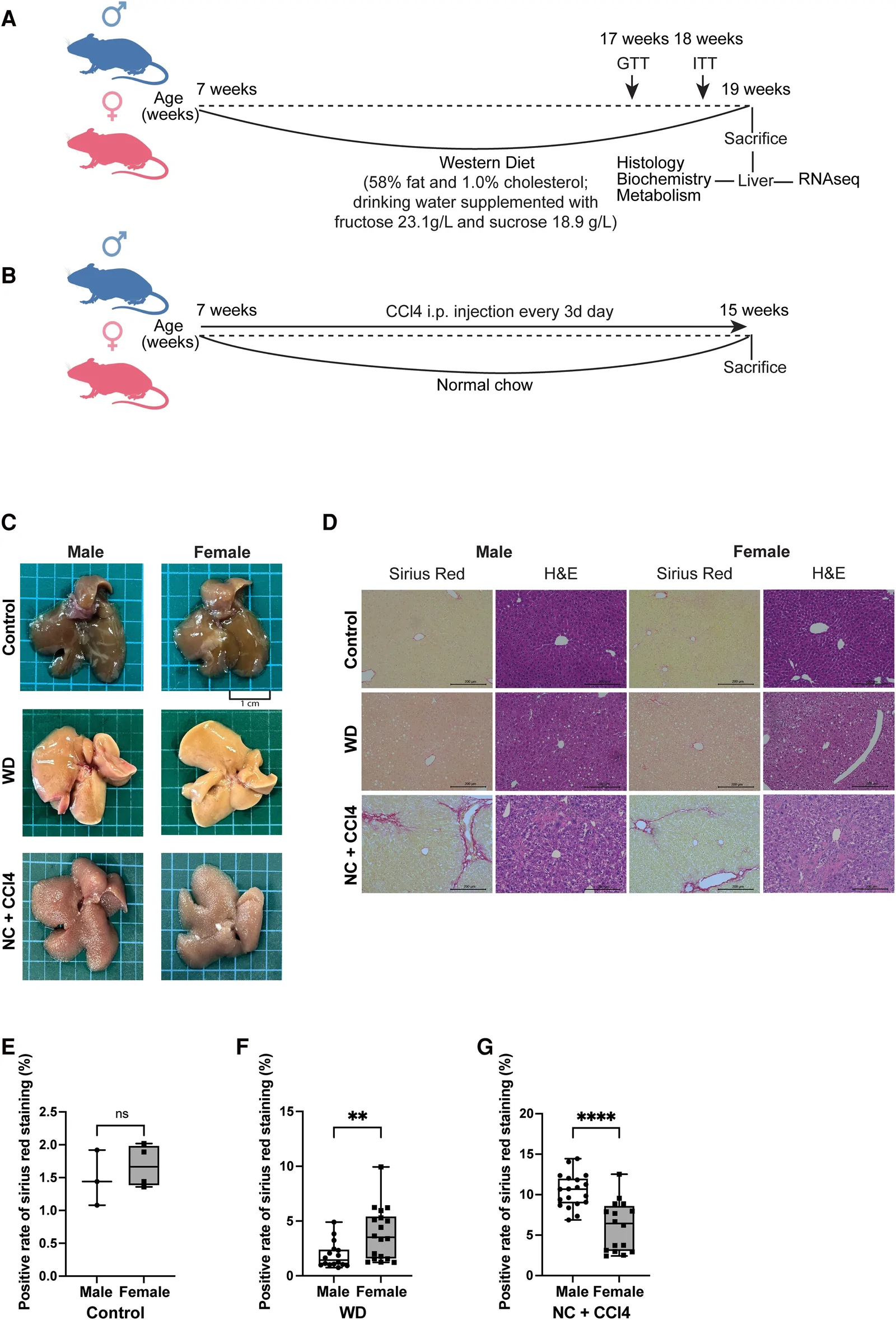
Representative morphologic and histologic features of the liver. (A and B) Study design. Created in BioRender. Khusnullina, A. (2026) https://BioRender.com/jp3vvs3. (C) Liver appearance. Scale bar: 1 cm. (D) Sirius Red staining. Scale bar: 200 µm. (E) Sirius Red–positive area in the control group. (F) Sirius Red–positive area in the WD group. (G) Sirius Red–positive area in the NC + CCl_4_ group. Control group: male, *n* = 3; female, *n* = 4. WD group: male, *n* = 17; female, *n* = 18. NC + CCl_4_ group: male, *n* = 19; female, *n* = 16. **P < .01, and ****P < .0001. ns, not significant; NC, normal chow; WD, Western diet; CCl_4_, carbon tetrachloride.

#### Ovariectomy

Bilateral ovariectomy was performed in female mice (*n* = 18) under isoflurane anesthesia according to a previously described protocol (14). After a recovery period of 1 week, ovariectomized (OVX) females, intact females, and males were fed the WD for 12 weeks or injected with CCl_4_, respectively. At the end of the intervention, liver fibrosis was assessed by Sirius Red staining. Because circulating estrogen levels were not measured and estrogen replacement experiments were not performed, the OVX experiment was interpreted as exploratory. Sham-operated remaining mice underwent the same skin and muscle incision without ovary removal.

#### Chemical-induced fibrosis model (carbon tetrachloride)

In a separate experiment designed to induce liver inflammation and fibrosis, mice were maintained on normal chow (MF; Oriental Yeast Co., Ltd) with regular drinking water and received intraperitoneal injections of carbon tetrachloride (CCl_4_) every 3 days for 8 weeks. CCl_4_ was diluted in olive oil (1:9, v/v), and the administered dose was 0.9 μL/g body weight (15). Sample sizes were *n* = 19 male and *n* = 16 female mice (Fig. 1B) and *n* = 9 OVX female mice (Fig. S2A (13)).

#### Sample collection

At the time of sacrifice, mice were deeply anesthetized with isoflurane. Urine samples were collected immediately before euthanasia. For tissue collection, mice were perfused via the portal vein with cold phosphate-buffered saline, and livers were excised. Liver tissues were snap-frozen in liquid nitrogen and stored at −80 °C for molecular and biochemical analyses or fixed in 4% paraformaldehyde for histological analyses.

### Histological and biochemical analyses

#### Liver histology and fibrosis assessment

Liver tissues fixed in 4% paraformaldehyde were paraffin-embedded and sectioned at 4 μm. To evaluate liver histology, sections were subjected to hematoxylin and eosin staining and Sirius Red staining. Liver histology was scored blindly by a single pathologist (K.H.) for histologic evaluation. The degree of fatty change, hepatocellular ballooning, and lobular inflammation were assessed according to the criteria established by the NASH Clinical Research Network (16). The Sirius Red–positive area was quantified as a measure of collagen deposition using QuPath software (version 0.3.2) on whole-slide images.

#### Immunohistochemistry

Macrophage infiltration was assessed by immunofluorescence staining for F4/80 (Cell Signaling Technology; cat. no. 70076; RRID: AB_2799771), as previously described (17). Paraffin sections were deparaffinized and rehydrated, followed by antigen retrieval in sodium citrate buffer (10 mM sodium citrate, 0.05% Tween-20, pH 6.0) using an autoclave (121 °C, 10 minutes). Sections were permeabilized with 0.3% Triton X-100 and blocked with Blocking One Histo (Nacalai Tesque), then incubated with the primary antibody overnight at 4 °C. After washing, sections were incubated with an Alexa Fluor 488–conjugated anti-rabbit IgG secondary antibody, and nuclei were counterstained with DAPI (VECTASHIELD mounting medium). Images were acquired using a fluorescence microscope (BZ-X810; KEYENCE), and positive cells were quantified.

#### Biochemical and oxidative stress assays

Blood glucose was measured using a portable glucometer (GLUCOCARD G+ Meter; ARKRAY, Kyoto, Japan), according to the manufacturer's instructions. Hepatic lipid peroxidation was assessed by measuring thiobarbituric acid-reactive substances (TBARS) using a commercial kit (TBARS Assay Kit; Cayman Chemical, Ann Arbor, MI, United States) according to the manufacturer's protocol. TBARS levels were calculated from the kit standard curve provided with the kit and reported as malondialdehyde-equivalent values. Oxidative DNA damage was evaluated by measuring urinary 8-hydroxy-2′-deoxyguanosine (8-OHdG) using a commercial ELISA kit (JaICA, Shizuoka, Japan) utilizing an anti-8-OHdG monoclonal antibody (clone N45.1; Japan Institute for Control of Aging Cat# MOG-020P, RRID: AB_3096485), according to the manufacturer's instructions. Concentrations were determined from a standard curve and reported as absolute values.

### Transcriptomic analysis

#### Bulk RNA sequencing

Bulk RNA sequencing (RNA-seq) was performed using liver tissue collected from the left lobe of mice fed a WD for 12 weeks (*n* = 4 independent mice per group). Total RNA was extracted by the service provider using the QuickGene-AutoS RNA Cultured Cell Kit (KURABO), according to the manufacturer's instructions; DNase treatment was not performed. RNA quality and concentration were assessed using an Agilent Bioanalyzer, including measurement of the RNA integrity number. Strand-specific mRNA libraries were prepared from 200 ng input RNA using the TruSeq Stranded mRNA Library Prep Kit (Illumina) with poly(A) selection. Libraries were indexed using IDT for Illumina TruSeq RNA UD Indexes (Illumina), pooled in proportion to the desired data yield, and sequenced on a NovaSeq X Plus platform (Illumina) in paired-end 101 bp mode (101 + 101). The target sequencing depth was 30 million paired-end reads per sample. For primary processing, reads were aligned to the mouse reference genome (GRCm38) using HISAT2 (v2.1.0). Gene-level counts were generated with featureCounts (v2.0.0) using the National Center for Biotechnology Information annotation (GCA_000001635.6). In addition, fragments per kilobase of transcript per million mapped reads (FPKM) values were calculated using Cuffdiff (v2.2.1). Differential expression analysis between groups was performed by the service provider using a log-transformed *t*-test on normalized counts without multiple-testing correction. Volcano plots were generated in R (v4.2.2) using ggplot2 (v3.4.2), ggpubr (v0.5.0), and ggrepel (v0.9.2). Genes meeting the significance threshold (*P* < .05) and exhibiting an absolute log_2_-fold change >2.0 were highlighted.

#### Integration with public single-cell RNA-seq datasets

A publicly available liver single-cell RNA-seq (scRNA-seq) dataset from WD-fed mice was obtained from GEO (GSE156057; GSM4721497; liver CD45^+^ cells, WD for 12 weeks) (11). The processed 10× Genomics HDF5 matrix was imported into Seurat using Read10X_h5, and a Seurat object was created, retaining cells that expressed >200 features. Quality-control filtering excluded cells with >5000 detected features or a mitochondrial transcript proportion ≥15%. Data were log-normalized, highly variable features were selected (2000 genes), and scaled values were computed while regressing out cell-cycle scores (S and G2M). Principal component analysis was performed, followed by graph-based clustering (Louvain; resolution 0.5) and uniform manifold approximation and projection (UMAP) visualization using the first 20 principal components. Differential expression results from bulk liver RNA-seq (male vs female comparison) were converted to a signed-rank metric and restricted to genes shared with the scRNA-seq dataset. For each scRNA-seq cluster, cluster-specific gene sets were defined using the top upregulated marker genes (top 200 genes per cluster; adjusted *P* < .05; positive log_2_-fold change) identified by FindAllMarkers. Gene set enrichment analysis was then performed using fgsea (multilevel implementation) to evaluate enrichment of the bulk RNA-seq signature across cluster gene sets. Enrichment results were summarized by normalized enrichment scores and false discovery rate–adjusted *P-*values.

#### Single-cell RNA sequencing analysis

Mouse liver tissues from male (*n* = 3) and female (*n* = 3) C57BL/6J mice collected at 4 weeks of WD feeding were snap-frozen in liquid nitrogen immediately after sacrifice and submitted to an external service provider for single-cell gene expression profiling. Single-cell RNA-seq libraries were prepared using the Chromium GEM-X Single Cell Gene Expression Flex platform (10× Genomics). Fixed single-cell suspensions, rather than nuclei, were processed using the 10× Fixation Kit and the Chromium GEM-X Flex Sample Preparation v2 Kit, according to the manufacturer's protocol. Libraries were generated with Chromium Single Cell Flex Gene Expression v1—GEM-X reagents and the Chromium Mouse Transcriptome Probe Set v1.1.1, GRCm39-2024-A. Raw sequencing data were processed using Cell Ranger with the corresponding GRCm39-2024-A mouse reference and probe set. Quality-control metrics were obtained from the Cell Ranger web summary files. Transcriptomic data analysis was performed using Seurat (v4.3.0). Cells with 200 to 5500 detected genes and <15% mitochondrial transcripts were retained for downstream analysis. The data were normalized and scaled, and S-phase/G2M-phase scores were regressed out to reduce cell-cycle effects. Cross-dataset integration used Seurat's anchor-based workflow (SelectIntegrationFeatures, FindIntegrationAnchors, and IntegrateData). Dimensionality reduction was performed using PCA, with cell clusters visualized via UMAP. The public scRNA-seq dataset and our newly generated scRNA-seq dataset were analyzed separately and were not computationally integrated into a single merged Seurat object. Each dataset underwent independent quality control, normalization, cell-type annotation, and dimensionality reduction (UMAP). At the initial exploratory stage, the public scRNA-seq dataset was integrated with our bulk RNA-seq dataset to broadly identify potential pathways, cell-type candidates, and markers involved in liver pathology. Subsequently, to independently validate these candidate pathways at single-cell resolution, we generated our own distinct scRNA-seq dataset. This newly generated scRNA-seq dataset was analyzed separately (without merging into the public single-cell object).

### Molecular validation

#### Protein extraction and immunoblotting

Liver tissues were homogenized in ice-cold radioimmunoprecipitation assay buffer supplemented with a protease inhibitor cocktail. Homogenates were clarified by centrifugation, and protein concentrations were determined using a bicinchoninic acid assay (Thermo Fisher Scientific). Equal amounts of protein (100 µg per lane) were mixed with 4× Laemmli reducing sample buffer, heat-denatured, separated on 12.5% polyacrylamide gels (e-PAGEL; ATTO), and transferred to polyvinylidene fluoride membranes (Millipore). Membranes were blocked in 3% nonfat dry milk in Tris-buffered saline containing 0.1% Tween-20 (TBS-T) at 4 °C, then incubated overnight at 4 °C with primary antibodies against 4-hydroxynonenal (4-HNE; rabbit polyclonal; Abcam Cat# ab46545, RRID: AB_722490; 1:300) and β-actin (mouse monoclonal; Sigma-Aldrich Cat# A5441, RRID: AB_476744; 1:10 000). After washing with TBS-T, membranes were incubated with IRDye-conjugated secondary antibodies (LI-COR) diluted 1:10 000: anti-rabbit IRDye 680RD (LICORbio Cat# 926-68071, RRID: AB_10956166) and anti-mouse IRDye 800CW (LICORbio Cat# 926-32212, RRID: AB_621847). Signals were detected using an Odyssey Infrared Imaging System (LI-COR Biosciences), and band intensities were quantified and normalized to β-actin.

### Statistical analysis

Statistical analyses were performed using GraphPad Prism version 9.3.0 (GraphPad Software, La Jolla, CA, United States). Data were assessed for normality and, when appropriate, for equality of variances was evaluated as appropriate. For comparisons between 2 groups, an unpaired 2-tailed Student *t*-test was applied for normally distributed data with equal variances; otherwise, the Mann–Whitney *U* test was used. For comparisons involving more than 2 groups, 1-way analysis of variance followed by the appropriate multiple-comparisons test was employed for parametric data, whereas the Kruskal–Wallis test followed by Dunn multiple-comparisons test was used for nonparametric data. Data are presented as mean ± SEM. A 2-sided *P*-value <.05 was considered statistically significant.

## Results

### Sex-specific associations between dietary nutrient density and ultrasound-defined liver steatosis in the Shika study

In sex-stratified analyses of the Shika study, we observed clear sexual dimorphism in the relationship between energy-adjusted nutrient density and liver steatosis. Among men, dietary profiles—including dietary cholesterol, total fat, saturated fat, sucrose, and total carbohydrate densities—did not differ significantly between participants with and without steatosis (all *P* > .05). Similarly, neither alcohol consumption nor total energy intake differed significantly by steatosis status (Table 1). In an exploratory analysis of carbohydrate composition, the sucrose-to-total carbohydrate ratio was also comparable between groups (*P* = .996).

**Table 1:** Energy-adjusted nutrient intakes and carbohydrate composition by liver steatosis status in men

Factor	No steatosis (*n* = 149)	Steatosis (*n* = 102)	*P*-value
Alcohol (g/day)	3.51 (0.00-16.71)	1.12 (0.00-12.11)	.068
Total energy (kcal/day)	1992.82 (1626.56-2426.73)	2045.66 (1525.22-2289.44)	.645
Protein (g/1000 kcal)	36.87 (33.12-40.92)	35.33 (31.22-42.00)	.336
Total fat (g/1000 kcal)	26.40 (22.55-29.62)	27.15 (22.13-30.39)	.707
Carbohydrate (g/1000 kcal)	142.63 (131.15-151.89)	142.02 (128.64-155.55)	.662
Saturated fat (g/1000 kcal)	6.65 (5.60-8.13)	6.83 (5.74-8.26)	.523
Dietary cholesterol (mg/1000 kcal)	183.48 (141.67-243.02)	187.07 (144.68-232.96)	.9
Sucrose (g/1000 kcal)	5.52 (3.12-8.68)	5.46 (3.18-9.45)	.85
Sucrose-to-total carbohydrate ratio (%)	4.02 (2.37-5.92)	3.90 (2.20-6.08)	.996

Values are given as median (interquartile range). Nutrients are energy-adjusted using the nutrient density method (per 1000 kcal), except for alcohol intake (g/day) and total energy intake (kcal/day). The sucrose-to-total carbohydrate ratio (%) was calculated as sucrose intake divided by total carbohydrate intake × 100. *P*-values were calculated using the Mann–Whitney *U* test. Statistical significance was defined as *P* < .05.

In contrast, women exhibited a distinct dietary signature associated with steatosis. Specifically, liver steatosis in women was associated with significantly higher densities of dietary cholesterol (*P* = .012), total fat (*P* = .004), and saturated fat (*P* = .009), alongside a lower total carbohydrate density (*P* = .017; Table 2). Although absolute sucrose density did not differ significantly (*P* = .121), the sucrose-to-total carbohydrate ratio was significantly higher in women with steatosis (*P* = .041), suggesting a shift in carbohydrate composition rather than a higher absolute sucrose intake. Alcohol consumption and total energy intake remained comparable between groups (Table 2).

**Table 2:** Energy-adjusted nutrient intakes and carbohydrate composition by liver steatosis status in women

Factor	No steatosis (*n* = 289)	Steatosis (*n* = 117)	*P*-value
Alcohol (g/day)	0.00 (0.00-0.34)	0.00 (0.00-0.59)	.383
Total energy (kcal/day)	1603.91 (1261.79-1934.32)	1554.03 (1326.98-1885.83)	.7
Protein (g/1000 kcal)	39.46 (34.68-44.74)	39.02 (34.37-44.80)	.994
Total fat (g/1000 kcal)	28.60 (24.25-33.09)	30.88 (26.25-35.16)	.004^a^
Carbohydrate (g/1000 kcal)	140.57 (128.31-152.97)	135.99 (122.75-150.64)	.017^a^
Saturated fat (g/1000 kcal)	7.49 (6.25-8.90)	8.06 (6.84-9.39)	.009^a^
Dietary cholesterol (mg/1000 kcal)	197.71 (157.06-248.83)	219.41 (174.05-259.02)	.012^a^
Sucrose (g/1000 kcal)	6.57 (3.77-9.74)	6.76 (4.37-11.34)	.121
Sucrose-to-total carbohydrate ratio (%)	4.69 (2.65-7.13)	5.20 (3.54-8.26)	.041^a^

Values are given as median (interquartile range). Nutrients are energy-adjusted using the nutrient density method (per 1000 kcal), except for alcohol intake (g/day) and total energy intake (kcal/day). The sucrose-to-total carbohydrate ratio (%) was calculated as sucrose intake divided by total carbohydrate intake × 100. *P*-values were calculated using the Mann–Whitney *U* test. ^a^Statistically significant, *P* < .05.

To better characterize the clinical background of the cohort, we added sex-stratified baseline characteristics according to ultrasound-defined liver steatosis status (Table S1 (13)). Because direct menopausal status was unavailable, age ≥55 years was used as an approximate proxy for postmenopausal age. Overall, 302 of 406 women (74.4%) were aged ≥55 years, suggesting that the female cohort largely represented a postmenopausal-age population. In an exploratory analysis restricted to participants with ultrasound-defined steatosis, age and FIB-4 values were comparable between women and men. FIB-4 did not differ between women and men with steatosis (women, 1.13 [0.82-1.49], *n* = 117; men, 1.14 [0.81-1.55], *n* = 102; *P* = .968), suggesting a comparable estimated fibrosis burden between sexes within the steatosis subgroup (Table S2 (13)). To address potential confounding by clinical variables, we performed additional multivariable logistic regression analyses in women. Each nutrient variable was entered into a separate model adjusted for age, alcohol intake, total energy intake, and HbA1c. Higher total fat, saturated fat, dietary cholesterol density, and sucrose-to-total carbohydrate ratio remained associated with ultrasound-defined liver steatosis in women (Table S3 (13)). After additional adjustment for BMI, 3 nutrient variables remained significantly associated with ultrasound-defined liver steatosis in women, while the sucrose-to-total carbohydrate ratio showed a borderline association (Table S3 (13)). After further adjustment for smoking status, all 4 nutrient variables remained significantly associated with ultrasound-defined liver steatosis (Table S3 (13)). In addition, we performed a sensitivity analysis restricted to women aged ≥55 years; we used the most fully adjusted model, including age, alcohol intake, total energy intake, HbA1c, BMI, and smoking status. Total fat, saturated fat, and dietary cholesterol remained significantly associated with ultrasound-defined liver steatosis in this subgroup. The sucrose-to-total carbohydrate ratio showed a consistent direction of association but did not reach conventional statistical significance (Table S4 (13)).

Collectively, these findings suggest that the diet–steatosis relationship is strongly sex-dependent, with identifiable dietary risk factors emerging specifically in women. To model these female-specific dietary risk factors, characterized by higher fat and cholesterol density together with a shift in carbohydrate composition toward sucrose, we selected a cholesterol-containing WD supplemented with fructose–sucrose drinking water for subsequent mouse experiments (Table S5 (13)). This reverse-translational design enabled us to test sex-dependent liver responses under a nutritionally relevant challenge aligned with the human epidemiological study.

### Female mice exhibit greater liver fibrosis than male mice in a nutrition-driven model, distinct from toxin-induced injury

To assess sex differences in nutrition-driven liver injury, 7-week-old male and female C57BL/6J mice were fed a WD for 12 weeks (Fig. 1A). Liver fibrosis was assessed by Sirius Red staining to quantify collagen deposition. Although both sexes developed fibrosis, the response was sexually dimorphic, with a female bias. Quantification of the Sirius Red–positive area demonstrated a significantly higher fibrotic burden in females compared with males (Fig. 1C, 1D, and 1F), while there was no difference in the control group fed normal chow between sexes (Fig. 1C-1E). Moreover, there was no significant difference in H&E results (Table S6 (13)). Interestingly, male mice exhibited a more severe metabolic phenotype than females, including higher body weight (Fig. S1A (13)) and blood glucose (Fig. S1B (13)), as well as greater glucose and insulin intolerance (Fig. S1C-S1G (13)). These differences were not associated with the liver weight/body weight ratio (Fig. S1H (13)). Importantly, this metabolic pattern did not parallel the fibrosis phenotype. Specifically, female mice developed severe liver fibrosis despite exhibiting a milder systemic metabolic profile compared with males. Moreover, estimated food intake and total caloric intake did not significantly differ between male and female mice, suggesting that differences in caloric consumption alone are unlikely to explain the female-biased fibrotic phenotype (Table S7 (13)).

To determine whether female susceptibility was specific to nutritional stress or a general predisposition to liver injury, we compared these results with a standard hepatotoxic model. In a separate cohort treated with CCl4 for 8 weeks (Fig. 1B), this sex difference was reversed: males exhibited significantly greater fibrosis than females (Fig. 1C, 1D, and 1F).

Under WD feeding, both intact and OVX females developed significantly greater Sirius Red–positive fibrosis compared with males (Fig. S2A-S2C (13)). In contrast, in the chemically induced model, OVX exacerbated fibrosis relative to intact females (Fig. S2B and S2D (13)). Crucially, these findings do not imply that ovarian hormones are inherently pathogenic nor do they establish estrogen deficiency as a primary driver of metabolic fibrosis. Our experimental data in OVX female mice demonstrated that female-biased fibrotic susceptibility persists independently of ovarian function, supporting the biological relevance of our clinical observations.

Given that the WD model, unlike the CCl_4_ model, captured the female-biased susceptibility implicated in our human observations, we identified it as the relevant platform to investigate nutrition-driven pathogenesis. Collectively, these results indicate that WD feeding enhances liver fibrosis in female mice relative to males, whereas the CCl_4_-based strong hepatotoxic fibrosis model yields the opposite sex pattern.

### Bulk RNA-seq reveals sex-biased hepatic transcriptional programs after WD feeding

To characterize sex-dependent transcriptional responses, we performed bulk RNA-seq on livers from mice fed a WD for 12 weeks (Fig. 2A). Differential expression analysis identified distinct gene signatures enriched in each sex.

**Figure 2:**
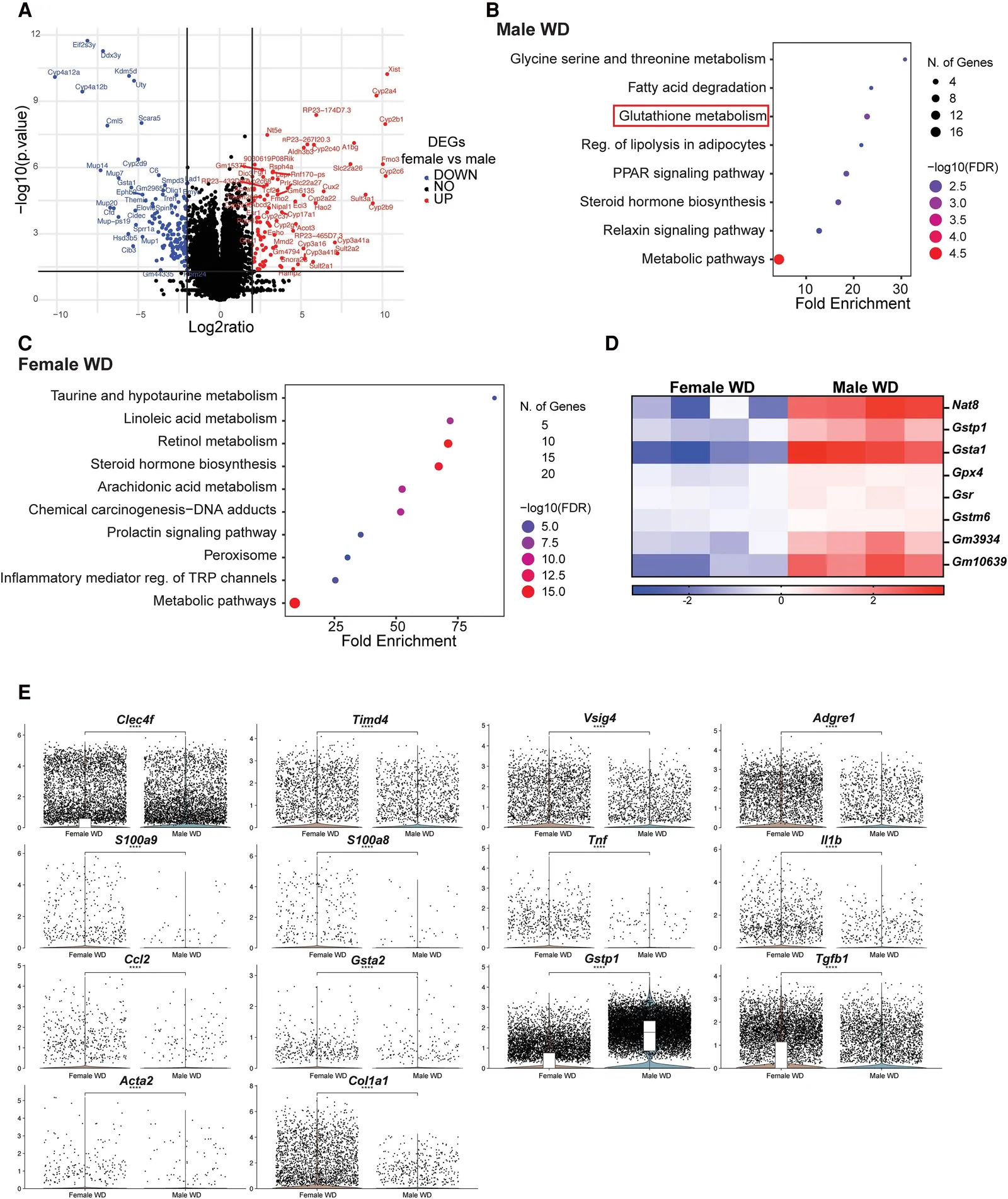
RNA sequencing results and hepatic expression of genes involved in liver inflammation and significantly altered pathways in male and female livers. (A) Volcano plot of differently expressed genes (DEGs) in female vs male mice fed WD for 12 weeks, showing log_2_-fold change (FC) thresholds of ±2.0. Blue (left region) indicates downregulated genes, and red (right region) indicates upregulated genes. The 2 vertical black lines represent log_2_FC cut-offs (>2.0 and <−2.0), and the horizontal black line indicates an adjusted *P*-value <.05. Genes in the upper left region (blue) are downregulated in female mice and upregulated in male mice (log_2_FC < −2.0; adjusted *P* < .05). Genes in the upper center region (black) show no substantial change in expression (−2.0 < log_2_FC < 2.0) but have an adjusted *P*-value <.05. Genes in the upper right region (red) are upregulated in female mice and downregulated in male mice (log_2_FC > 2.0; adjusted *P* < .05). Genes below the horizontal black line are not statistically significant (adjusted *P* > .05) and are considered unchanged. (B and C) KEGG pathway analysis based on significantly higher expressed genes in male and female mice fed WD for 12 weeks, respectively. (D) Heat map of genes involved in glutathione metabolism among male and female mice fed WD for 12 weeks. Expression values are shown as Cuffdiff-derived normalized FPKM values (log_2_-transformed for visualization; row scaling applied where indicated). (E) Violin plot of Kupffer cells marker genes: *Clec4f*, *Timd4*, *Vsig4*, and *Adgre1*; MoM marker genes: *S100a9* and *S100a8*; inflammatory-associated marker genes: *Tnf*, *Ccl2*, and *Il1b*; glutathione metabolism marker genes: *Gsta2* and *Gstp1*; and fibrosis marker genes: *Tgfb1, Acta2*, and *Col1a1*. Statistical analyses were performed using the Wilcoxon rank-sum test. RNA sequencing analyses were performed using the 12-week WD-fed mouse model (*n* = 4 per group). DEG, differently expressed gene; WD, Western diet.

Male mice exhibited a coordinated upregulation of genes associated with the antioxidant pathway. Specifically, males showed higher expression of several downstream targets involved in glutathione metabolism, including *Nat8*, *Gstp1*, *Gsta1*, *Gpx4*, *Gsr*, *Gstm6*, *Gm3934*, and *Gm10639* (Fig. 2A and 2B). Heat map visualization confirmed the robust enrichment of these detoxification genes in males relative to females (Fig. 2D, Table S8 (13)). In contrast, female mice showed increased expression of genes involved in arachidonic acid metabolism and fatty acid oxidation, including cytochrome P450 enzymes (C*yp2b10*, *Cyp2b13*, *Cyp2c40*, and *Cyp4a14*) and acyl-CoA thioesterases (*Acot1*, *Acot3*, and *Acot4*; Fig. 2A and 2C).

To validate these transcriptomic profiles, we compared Kupffer cell marker genes: *Clec4f*, *Timd4*, *Vsig4*, and *Adgre1*; MoM marker genes: *S100a9* and *S100a8*; inflammatory-associated marker genes: *Tnf*, *Ccl2*, and *Il1b*; glutathione metabolism marker genes: *Gsta2* and *Gstp1*; and fibrosis marker genes: *Tgfb1*, *Acta2*, and *Col1a1* genes expression based on scRNA-seq results (Fig. 2E). Consistent with the RNA-seq data, males showed higher expression of *Gsta2* and *Gstp1*, whereas females exhibited higher expression of *Tnf*, *Il1b*, *Ccl2*, *S100a9*, *S100a8*, *Adgre1*, *Tgfb1*, *Acta2*, and *Col1a1* (Fig. 2E). Each sample's quality control and sequencing metrics are shown in Table S9 (13).

### Females exhibit greater oxidative damage than males under WD feeding

We next evaluated oxidative injury by quantifying lipid peroxidation and DNA damage markers. Hepatic TBARS levels were significantly higher in females than in males (Fig. 3A). Consistent with this finding, immunoblot analysis revealed increased levels of hepatic 4-HNE adducts in females relative to males (Fig. 3B and 3C). Additionally, urinary 8-OHdG levels were significantly higher in females than in males (Fig. 3D), indicating increased systemic oxidative stress in the female mice.

**Figure 3:**
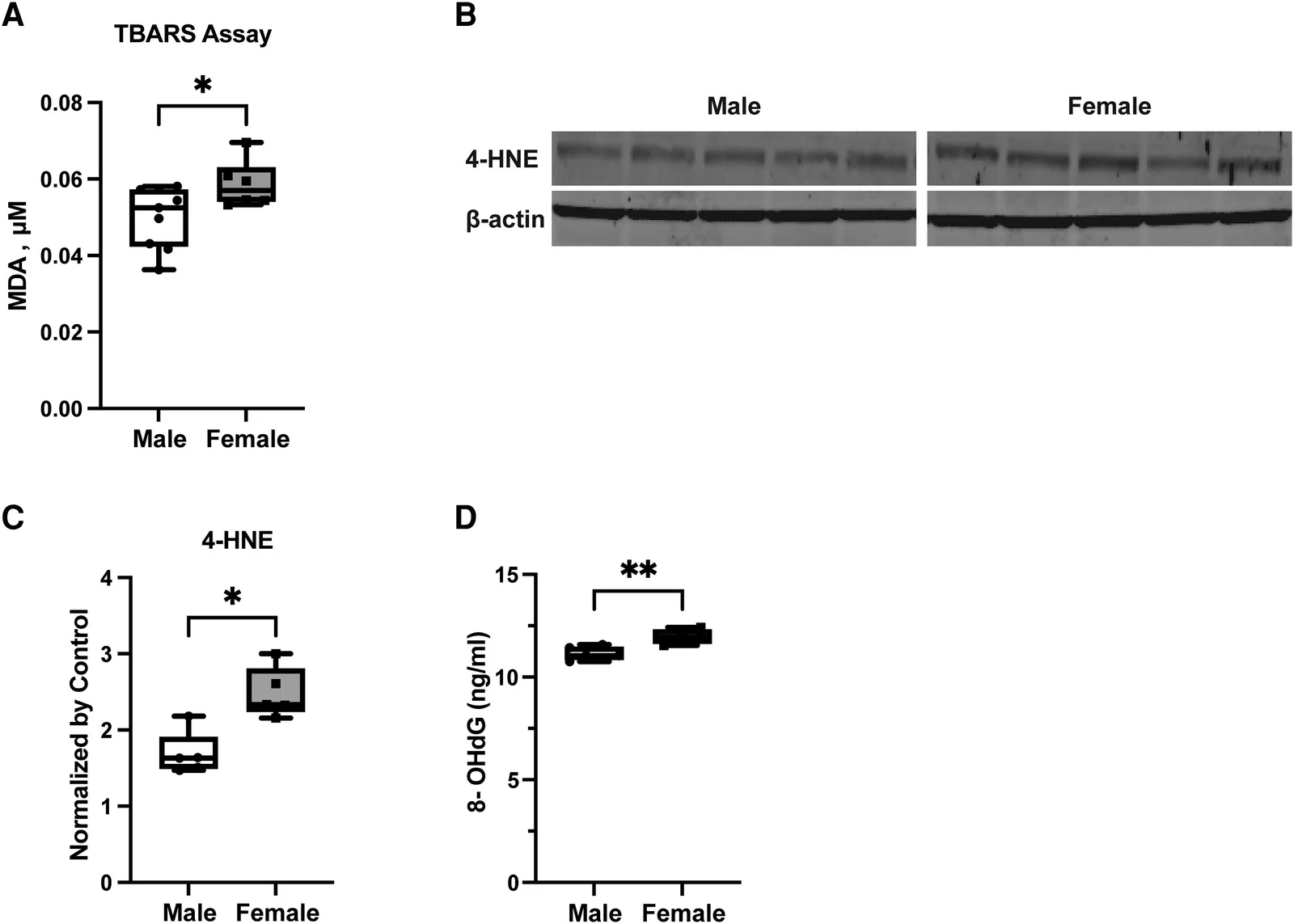
Oxidative stress markers. (A) TBARS assay showing MDA expression level in the liver. (B and C) Immunoblots of 4-HNE-modified proteins. Quantification was normalized to β-actin levels (control NC mice: *n* = 3; female WD mice: *n* = 5; male WD mice: *n* = 5). (D) 8-OhdG expression levels in urine measured by ELISA. *P < .05, **P < .01. WD, Western diet; NC, normal chow; 4-HNE, 4-hydroxynonenal; TBARS, thiobarbituric acid reactive substances; MDA, malondialdehyde; 8-OhdG, 8-hydroxy-2′-deoxyguanosine.

### Female mice exhibit increased hepatic macrophage accumulation and a Kupffer cell–skewed transcriptomic signature

To assess hepatic inflammation, we performed immunohistochemistry for the macrophage marker F4/80 (Fig. 4A). Quantification revealed a significantly higher number of F4/80-positive cells in females than in males (Fig. 4B).

**Figure 4:**
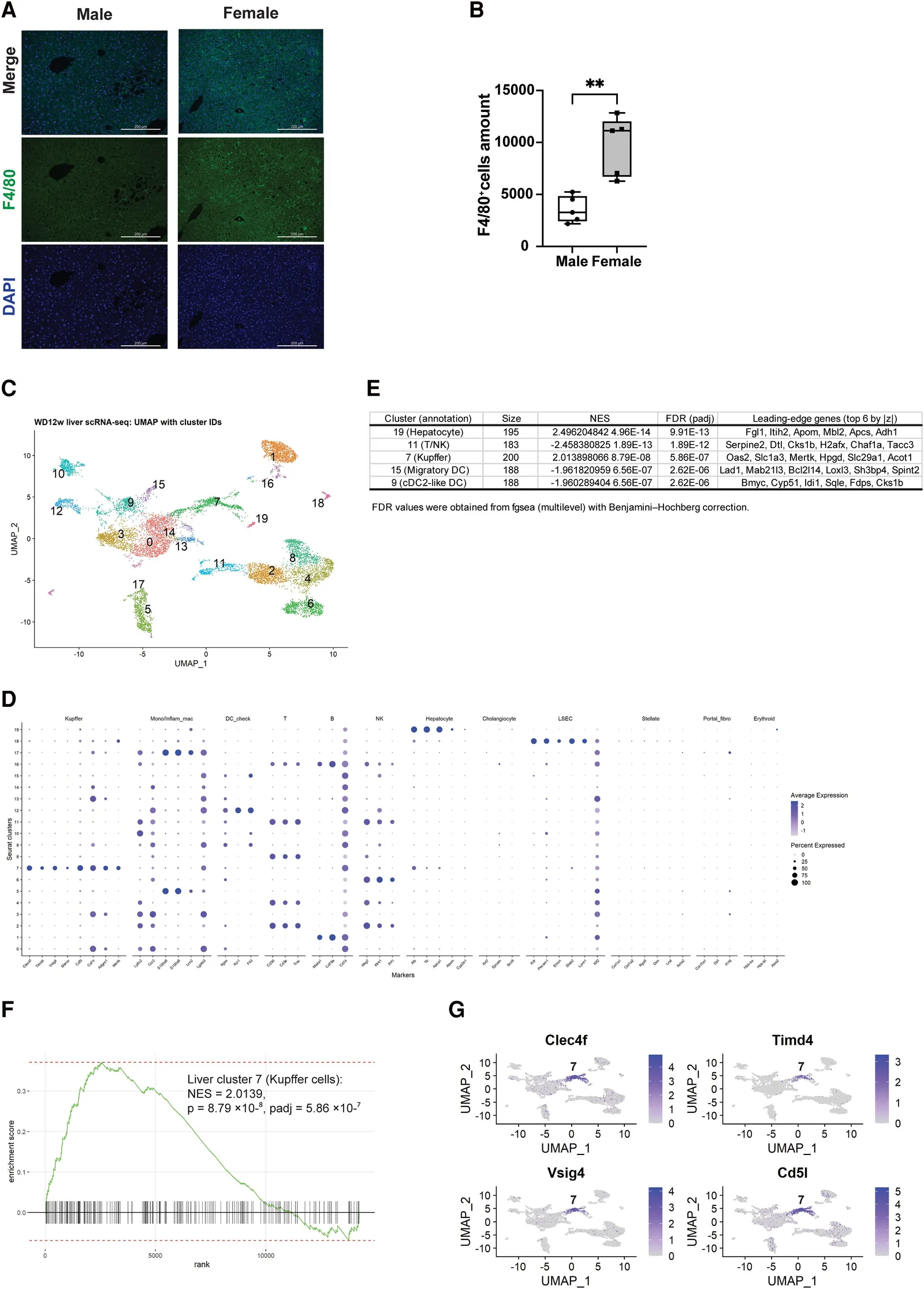
Top enriched liver cell clusters underlying sex-biased bulk liver expression. (A) Immunofluorescent staining of liver sections for F4/80 (pan-macrophage marker; green) with nuclear counterstaining by DAPI (4′,6-diamidino-2-phenylindole; blue). Scale bar: 200 µm. (B) Quantification of total F4/80 cells (male WD: *n* = 5; female WD: *n* = 5). (C) UMAP of the 12-week WD liver single-cell RNA-seq dataset with annotated Seurat clusters. (D) Dot plot of canonical markers used for cluster annotation (Cluster 7, Kupffer cells; Cluster 11, T/NK cells; Cluster 19, hepatocytes). (E) Summary table of fgsea results showing the top 5 enriched cluster marker gene sets based on the sex-biased bulk RNA-seq ranking (signed *z*-statistic). (F) Representative enrichment plot for the Cluster 7 (Kupffer cell) marker gene set. (G) UMAP feature plots of Kupffer cell markers (*Clec4f*, *Timd4*, *Vsig4*, *Cd5l*), highlighting their localization to Cluster 7. Positive NES indicates enrichment among genes upregulated in females in the bulk RNA-seq dataset, whereas negative NES indicates enrichment among genes upregulated in males. Leading-edge genes are defined as the top 6 genes ranked by |*z*| within the fgsea leading-edge subset. Data are presented as mean ± SEM. **P < .01, WD, Western diet; UMAP, uniform manifold approximation and projection; NES, normalized enrichment scores.

To further characterize hepatic immune-cell composition, we integrated the bulk liver RNA-seq data with a public liver scRNA-seq dataset (GSE156057). Seurat-based clustering of the public CD45^+^ liver scRNA-seq dataset resolved major hepatic immune-cell populations, including Kupffer cells, monocyte-derived/inflammatory macrophages, dendritic cells, T cells, B cells, and NK cells (Fig. 4C). Cell-type annotation was performed using a dot plot of canonical markers, identifying major immune populations—including Kupffer cells (Cluster 7), monocyte-derived/inflammatory macrophages, dendritic cells, T cells, B cells, and NK cells (Fig. 4D). Because this dataset was generated from CD45^+^ cells, we did not use it to draw conclusions regarding hepatocytes, cholangiocytes, liver sinusoidal endothelial cells, or hepatic stellate cells.

We next performed Gene Set Enrichment Analysis using the sex-biased bulk RNA-seq gene ranking to test enrichment across the scRNA-seq cluster marker gene sets. This analysis identified the 5 clusters most significantly enriched for sex-biased signatures (Fig. 4E). Notably, genes upregulated in the female bulk RNA-seq dataset were preferentially enriched in the Kupffer cell cluster (Cluster 7; Fig. 4F). Consistent with this enrichment, UMAP feature plots demonstrated that canonical Kupffer cell markers (*Clec4f*, *Timd4*, *Vsig4*, and *Cd5l*) were localized predominantly within Cluster 7 (Fig. 4G).

Together with the F4/80 immunostaining results, these findings support a female-skewed macrophage/Kupffer cell signature under WD feeding.

## Discussion

In this study, we employed a reverse-translational strategy to determine whether biological sex modifies susceptibility to nutrition-associated liver injury. Anchoring our experimental design in the Shika study, we first identified a female-specific dietary signature associated with liver steatosis, characterized by higher fat and cholesterol density and a shift in carbohydrate composition toward sucrose. To bridge the gap between these epidemiological signals and tissue-level pathology, we utilized a nutritionally relevant WD murine model. Our findings demonstrate that, under the same dietary conditions, female mice develop more pronounced liver fibrosis, oxidative injury, and macrophage accumulation than male mice. We suggest that the greater combined lipid, cholesterol, and sugar burden imposed by our dietary model may have attenuated the protective mechanisms typically observed in females reported in some previous models (12). Together, these results validate our bedside-to-bench approach and support the concept that sex is a critical biological modifier of the response to specific nutrient imbalances (13, 18).

A central insight from our work stems from the comparison between nutrition- and toxin-driven injury models. We observed a stark reversal in sex differences: while males were more susceptible to CCl_4_-induced injury, females were more susceptible to WD-induced fibrosis. This contrast underscores the context-dependent nature of sex effects and cautions against extrapolating findings from acute toxicity models to chronic metabolic diseases (19, 20). Crucially, detailed phenotyping revealed a dissociation between systemic metabolic status and liver pathology. Male mice exhibited a more severe metabolic syndrome phenotype, including greater obesity and insulin resistance, yet female mice developed more severe liver fibrosis. This observation is pivotal, suggesting that the female-biased progression to fibrosis is not simply parallel and not merely a secondary consequence of systemic metabolic dysfunction but rather stems from an intrinsic hepatic susceptibility to the specific nutritional challenge.

Our transcriptomic and biochemical analyses provide a mechanistic explanation for this female-specific vulnerability, centering on a “double hit” of compromised defense and enhanced toxicity. First, we identified a critical upstream divergence in the antioxidant pathway. Male livers exhibited significantly higher basal transcriptional activity governing the detoxification system. As this master upstream activation coordinates the constitutive expression of *Gsta* and *Gstm* family genes (21), this male-specific regulatory pattern likely orchestrates the robust upregulation of glutathione-mediated defenses observed in our study. Indeed, this response involves coordinated induction of *Nat8*, *Gstp1*, *Gsta1*, *Gpx4*, *Gsr*, *Gstm6*, *Gm3934*, and *Gm10639*. GPX4 acts as a first line of defense by directly reducing lipid hydroperoxides and preventing ferroptosis (22-24). Crucially, this mechanism is complemented by the induction of GSTA enzymes (eg, *Gsta1* and *Gsta2*), which function as the second line of defense by conjugating and detoxifying electrophilic byproducts, such as 4-HNE, that escape initial scavenging (25, 26). In contrast, female livers failed to mount this dual protective response and instead upregulated genes involved in microsomal ω-oxidation (*Cyp4a14*) and acyl-CoA hydrolysis (*Acot1*). Although potentially compensatory, these pathways may also be maladaptive. Microsomal CYP4A enzymes are established sources of reactive oxygen species (ROS) that promote lipid peroxidation in metabolic dysfunction–associated steatohepatitis (27). Similarly, ACOT1 activity can increase the pool of lipotoxic free fatty acids, thereby promoting inflammation and progression from steatosis to steatohepatitis (28). Together, these findings suggest that the female liver activates a metabolically toxic program while lacking a sufficiently robust antioxidant defense.

Consistent with this imbalance, females exhibited significantly higher levels of lipid peroxidation adducts (4-HNE) and DNA damage. Notably, this elevated oxidative stress likely acts as a common driver aggravating both inflammation and fibrosis. The accumulation of lipid peroxidation products, such as 4-HNE, can impair mitochondrial respiratory function and compromise fatty acid β-oxidation capacity (29, 30). Furthermore, oxidative stress can disrupt very-low-density lipoprotein assembly and secretion, thereby trapping lipids within hepatocytes (31, 32). Collectively, these findings lead us to hypothesize a potential feed-forward female-specific antioxidant insufficiency pathway: initial lipid accumulation generates ROS, which further inhibits lipid disposal pathways, exacerbating steatosis, while simultaneously triggering the inflammatory-fibrotic cascade. However, whether female-specific antioxidant insufficiency directly drives this process remains to be functionally validated.

Our integration of bulk RNA-seq with scRNA-seq data supports this cascade, localizing the female-biased inflammatory signature specifically to Kupffer cells. As Kupffer cells are typically activated by ROS and lipid peroxidation products to promote fibrogenesis (33, 34), our data support a sequential model: female-specific metabolic maladaptation leads to unchecked oxidative stress, which may promote hepatic lipid retention (31) and Kupffer cell activation, thereby linking steatosis-associated metabolic stress to fibrogenesis. In our study, we could not find differences in the MoM-related gene set comparing males and females. The major reason is that our fibrosis model, which shows fibrosis with slight steatosis, is different from the previous model. There remains a question about the relationship among the differences in the diet, macrophage type, and liver phenotype. Further investigations are required.

This study has several limitations. First, because the Shika study was cross-sectional, causal relationships between nutrient intake and liver steatosis could not be inferred. Second, liver steatosis was assessed by abdominal ultrasonography as a binary variable, and liver histology, elastography, and quantitative imaging-based assessment were unavailable; therefore, steatosis severity, steatohepatitis, and fibrosis stage could not be directly evaluated. Third, direct menopausal status and circulating sex hormone levels were unavailable, although most women were aged ≥55 years. Fourth, physical activity data were also not available. Additionally, although multivariable models supported associations between selected nutrient variables and ultrasound-defined steatosis in women, these findings should be interpreted as exploratory nutrient–steatosis associations rather than definitive causal evidence.

Moreover, the bulk RNA-seq analysis was exploratory and was performed to complement the histological and metabolic phenotypes. Because of the limited number of biological replicates, transcriptomic findings were interpreted cautiously and were reevaluated using false discovery rate (FDR) correction. Also, given the variations in experimental design and timing, the public dataset served strictly for exploratory cell-type and pathway annotation, whereas our newly generated dataset formed the primary basis for assessing the WD-fed mouse model.

Although we identified specific nutrient patterns in humans, the mouse diet was a fixed formulation. Finally, functional validation of the identified pathways is required to definitively establish causality. However, the strong concordance between transcriptomic signatures and oxidative/histological phenotypes provides robust support for our mechanistic hypothesis.

In conclusion, using an experimental model that incorporates the specific dietary risks identified in our human cohort, we demonstrate that biological sex fundamentally modifies the progression from simple steatosis to fibrosing metabolic dysfunction–associated steatohepatitis. Clinically, these findings have immediate translational implications: the stronger diet–steatosis association observed in women in the epidemiological analysis, together with their intrinsic hepatic vulnerability to nutrient imbalance demonstrated in the mouse model, suggests that dietary management may be particularly critical for female patients. Rigorous maintenance of dietary quality may be more critical for women than for men, serving as a primary strategy for both preventing MASLD onset and limiting disease progression. More broadly, this integrated framework indicates that therapeutic strategies should incorporate sex-specific considerations, emphasizing stringent nutritional intervention in women while exploring metabolic or cytoprotective targets in men.

## Data Availability

Original data generated and analyzed during this study are included in this published article or in the data repositories listed in the references.

## Abbreviations

4-HNE: 4-hydroxynonenal
8-OHdG: 8-hydroxy-2′-deoxyguanosine
BDHQ: Brief-type Self-administered Diet History Questionnaire
BMI: body mass index
HbA1c: hemoglobin A1c
MASLD: metabolic dysfunction–associated steatotic liver disease
OVX: ovariectomized
ROS: reactive oxygen species
TBARS: thiobarbituric acid-reactive substances
UMAP: uniform manifold approximation and projection
WD: Western diet

## References

[1] Huang DQ, Singal AG, Kono Y, Tan DJH, El-Serag HB, Loomba R. Changing global epidemiology of liver cancer from 2010 to 2019: NASH is the fastest growing cause of liver cancer. Cell Metab. 2022;34(7):969‐977.e2. 10.1016/j.cmet.2022.05.003 PMC9762323 35793659

[2] Hashimoto E, Tokushige K. Prevalence, gender, ethnic variations, and prognosis of NASH. J Gastroenterol. 2011;46(Suppl 1):63‐69. 10.1007/s00535-010-0311-8 20844903

[3] Balakrishnan M, Patel P, Dunn-Valadez S, et al Women have a lower risk of nonalcoholic fatty liver disease but a higher risk of progression vs men: a systematic review and meta-analysis. Clin Gastroenterol Hepatol. 2021;19(1):61‐71.e15. 10.1016/j.cgh.2020.04.067 PMC8796200 32360810

[4] Yang JD, Abdelmalek MF, Guy CD, et al Patient sex, reproductive status, and synthetic hormone use associate with histologic severity of nonalcoholic steatohepatitis. Clin Gastroenterol Hepatol. 2017;15(1):127‐131.e2. 10.1016/j.cgh.2016.07.034 PMC5161542 27523635

[5] Wegermann K, Garrett ME, Zheng J, et al Sex and menopause modify the effect of single nucleotide polymorphism genotypes on fibrosis in NAFLD. Hepatol Commun. 2021;5(4):598‐607. 10.1002/hep4.1668 PMC8034580 33860118

[6] Im YR, Hunter H, de Gracia Hahn D, et al A systematic review of animal models of NAFLD finds high-fat, high-fructose diets most closely resemble human NAFLD. Hepatology. 2021;74(4):1884‐1901. 10.1002/hep.31897 33973269

[7] Vacca M, Kamzolas I, Harder LM, et al An unbiased ranking of murine dietary models based on their proximity to human metabolic dysfunction-associated steatotic liver disease (MASLD). Nat Metab. 2024;6(6):1178‐1196. 10.1038/s42255-024-01043-6 PMC11199145 38867022

[8] Goto H, Yamamoto Y, Tsujiguchi H, et al Oxytocin receptor polymorphism is associated with sleep apnea symptoms. J Endocr Soc. 2024;9(1):bvae198. 10.1210/jendso/bvae198 PMC11590662 39606181

[9] Goto H, Yamamoto Y, Tsujiguchi H, et al Oxytocin receptor polymorphism rs53576 is linked to habitual alcohol and sucrose intake, as well as liver steatosis, in a general Japanese population cohort. J Nutr. 2026;156(3):101375. 10.1016/j.tjnut.2026.101375 41580086

[10] Kobayashi S, Honda S, Murakami K, et al Both comprehensive and brief self-administered diet history questionnaires satisfactorily rank nutrient intakes in Japanese adults. J Epidemiol. 2012;22(2):151‐159. 10.2188/jea.JE20110075 PMC3798594 22343326

[11] Remmerie A, Martens L, Thoné T, et al Osteopontin expression identifies a subset of recruited macrophages distinct from Kupffer cells in the fatty liver. Immunity. 2020;53(3):641‐657.e14. 10.1016/j.immuni.2020.08.004 PMC7501731 32888418

[12] Zhang H, Léveillé M, Courty E, Gunes A, Nguyen BN, Estall JL. Differences in metabolic and liver pathobiology induced by two dietary mouse models of nonalcoholic fatty liver disease. Am J Physiol Endocrinol Metab. 2020;319(5):E863‐E876. 10.1152/ajpendo.00321.2020 32924526

[13] Khusnullina A, Goto H, Kimura K, et al Supplemental data from Khusnullina et al., biological sex modifies the link between nutrient intake and MASLD: intrinsic oxidative vulnerability in females. Figshare repository. 10.6084/m9.figshare.31769353

[14] Luengo-Mateos M, González-Vila A, Torres Caldas AM, et al Protocol for ovariectomy and estradiol replacement in mice. STAR Protoc. 2024;5(1):102910. 10.1016/j.xpro.2024.102910 PMC10907206 38416648

[15] Abuduyimiti T, Goto H, Kimura K, et al Diabetes accelerates steatohepatitis in mice. Am J Pathol. 2024;194(5):693‐707. 10.1016/j.ajpath.2024.01.007 38309428

[16] Kleiner DE, Brunt EM, Van Natta M, et al Design and validation of a histological scoring system for nonalcoholic fatty liver disease. Hepatology. 2005;41(6):1313‐1321. 10.1002/hep.20701 15915461

[17] Tanida R, Goto H, Takayama H, et al LECT2 deletion exacerbates liver steatosis and macrophage infiltration in a male mouse model of LPS-mediated NASH. Endocrinology. 2024;165(7):bqae059. 10.1210/endocr/bqae059 38781447

[18] Arivazhagan L, Delbare S, Wilson RA, et al Sex differences in murine MASH induced by a fructose-palmitate-cholesterol-enriched diet. JHEP Rep. 2025;7(2):101222. 10.1016/j.jhepr.2024.101222 PMC11795143 39911943

[19] Nevzorova YA, Boyer-Diaz Z, Cubero FJ, Gracia-Sancho J. Animal models for liver disease – a practical approach for translational research. J Hepatol. 2020;73(2):423‐440. 10.1016/j.jhep.2020.04.011 32330604

[20] Meijnikman AS, Fondevila MF, Arrese M, Kisseleva T, Bataller R, Schnabl B. Towards more consistent models and consensual terminology in preclinical research for steatotic liver disease. J Hepatol. 2025;82(4):760‐766. 10.1016/j.jhep.2024.11.025 39581500

[21] Chanas SA, Jiang Q, McMahon M, et al Loss of the Nrf2 transcription factor causes a marked reduction in constitutive and inducible expression of the glutathione S-transferase Gsta1, Gsta2, Gstm1, Gstm2, Gstm3, and Gstm4 genes in the livers of male and female mice. Biochem J. 2002;365(Pt 2):405‐416. 10.1042/BJ20020320 PMC1222698 11991805

[22] Yang WS, SriRamaratnam R, Welsch ME, et al Regulation of ferroptotic cancer cell death by GPX4. Cell. 2014;156(1-2):317‐331. 10.1016/j.cell.2013.12.010 PMC4076414 24439385

[23] Forcina GC, Dixon SJ. GPX4 at the crossroads of lipid homeostasis and ferroptosis. Proteomics. 2019;19(18):e1800311. 10.1002/pmic.201800311 30888116

[24] Ingold I, Berndt C, Schmitt S, et al Selenium utilization by GPX4 is required to prevent hydroperoxide-induced ferroptosis. Cell. 2018;172(3):409‐422.e21. 10.1016/j.cell.2017.11.048 29290465

[25] Balogh LM, Atkins WM. Interactions of glutathione transferases with 4-hydroxynonenal. Drug Metab Rev. 2011;43(2):165‐178. 10.3109/03602532.2011.558092 PMC4464844 21401344

[26] Singhal SS, Singh SP, Singhal P, Horne D, Singhal J, Awasthi S. Antioxidant role of glutathione S-transferases: 4-hydroxynonenal, a key molecule in stress-mediated signaling. Toxicol Appl Pharmacol. 2015;289(3):361‐370. 10.1016/j.taap.2015.10.006 PMC4852854 26476300

[27] Gao H, Cao Y, Xia H, Zhu X, Jin Y. CYP4A11 is involved in the development of nonalcoholic fatty liver disease via ROS-induced lipid peroxidation and inflammation. Int J Mol Med. 2020;45(4):1121‐1129. 10.3892/ijmm.2020.4479 PMC7053872 32124935

[28] Pasini E, Baciu C, Angeli M, et al Acyl-CoA thioesterase 1 contributes to transition of steatosis to metabolic-associated steatohepatitis. Int J Hepatol. 2024;2024(1):5560676. 10.1155/2024/5560676 PMC11611406 39624175

[29] Chen J, Schenker S, Frosto TA, Henderson GI. Inhibition of cytochrome c oxidase activity by 4-hydroxynonenal (HNE). Role of HNE adduct formation with the enzyme subunits. Biochim Biophys Acta. 1998;1380(3):336‐344. 10.1016/s0304-4165(98)00002-6 9555085

[30] Andringa KK, Udoh US, Landar A, Bailey SM. Proteomic analysis of 4-hydroxynonenal (4-HNE) modified proteins in liver mitochondria from chronic ethanol-fed rats. Redox Biol. 2014;2:1038‐1047. 10.1016/j.redox.2014.09.006 PMC4297939 25454745

[31] Pan M, Cederbaum AI, Zhang YL, Ginsberg HN, Williams KJ, Fisher EA. Lipid peroxidation and oxidant stress regulate hepatic apolipoprotein B degradation and VLDL production. J Clin Invest. 2004;113(9):1277‐1287. 10.1172/JCI19197 PMC398425 15124019

[32] Pan M, Maitin V, Parathath S, et al Presecretory oxidation, aggregation, and autophagic destruction of apoprotein-B: a pathway for late-stage quality control. Proc Natl Acad Sci U S A. 2008;105(15):5862‐5867. 10.1073/pnas.0707460104 PMC2311371 18391222

[33] Xu GX, Wei S, Yu C, et al Activation of Kupffer cells in NAFLD and NASH: mechanisms and therapeutic interventions. Front Cell Dev Biol. 2023;11:1199519. 10.3389/fcell.2023.1199519 PMC10228659 37261074

[34] Wu H, Chen G, Wang J, Deng M, Yuan F, Gong J. TIM-4 interference in Kupffer cells against CCL4-induced liver fibrosis by mediating Akt1/Mitophagy signalling pathway. Cell Prolif. 2020;53(1):e12731. 10.1111/cpr.12731 PMC6985653 31755616

